# Supplementary material for: Neural network variational Monte Carlo for positronic chemistry
Source: Nat Commun. 2024 Jun 18;15:5214. doi: 10.1038/s41467-024-49290-1 (PMC11189582; doi:10.1038/s41467-024-49290-1)
Supplement: Supplementary file 1 — Supplementary Information [file 41467_2024_49290_MOESM1_ESM.pdf]

Supplementary Information: Neural network variational Monte Carlo for positronic chemistry

Gino Cassella[1]*, W.M.C. Foulkes[1], David Pfau[2,1], and James S. Spencer[2]
[1]*Dept. of Physics, Imperial College London, London SW7 2AZ, United Kingdom and*
[2]*DeepMind, London N1C 4DJ, United Kingdom*
(*g.cassella20@imperial.ac.uk)

VARIANCE MATCHING

Variance matching was utilised in the main text to calculate the positron binding energy for the sodium atom and the benzene molecule. Here we will describe the procedure for obtaining benzene's positron binding energy. We note that all variances are evaluated by freezing the wavefunction parameters and taking 10k samples of the local energy by MCMC sampling. The variance in the local energy is calculated using a reblocking procedure to account for sequential correlations in the MCMC sampling [1].

After a fixed number of optimisation steps (i.e. at the end of training), we calculate the variance of the local energy for the positronic ground-state wavefunction. We then calculate the variance of the local energy for the bare molecular ground-state

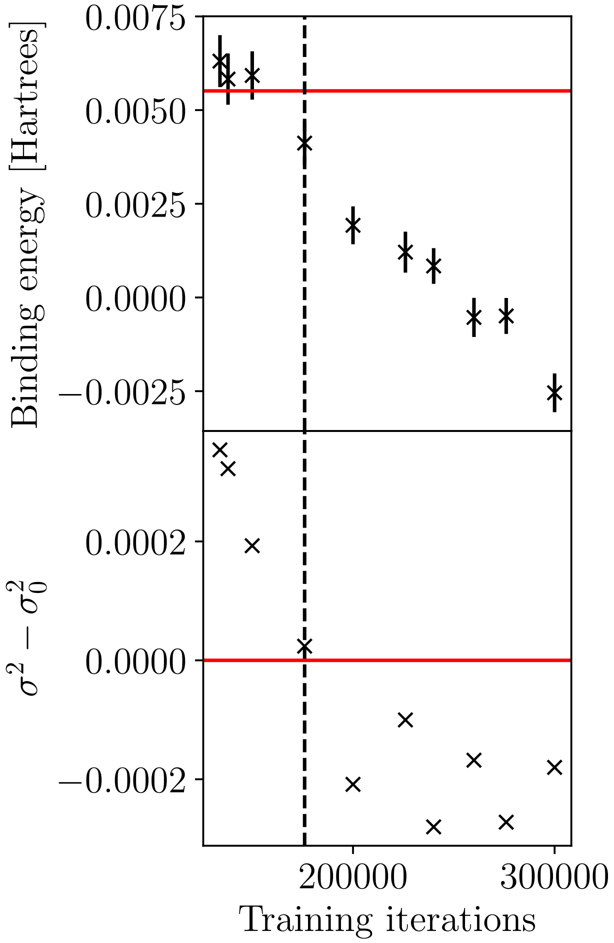

FIG. 1. **Demonstration of variance matching for the positron binding energy of benzene. Top**: Positron binding energy of the benzene molecule obtained using bare molecular ground-state energies obtained from checkpoints taken after varying training iterations. **Bottom**: Local energy variance difference between bare molecular checkpoints and positronic molecule at the end of training.

wavefunction at a series of regularly spaced checkpoints during training – approximately every 50k iterations from 150k to 300k. We refine the variance matching by calculating the variance between the two checkpoints where the difference crosses zero. We additionally calculate the variance at several other randomly chosen points in the range of interest to validate our implicit assumption that the variance is approximately monotonic. The closest variance match is obtained after 175k training iterations of the bare molecule. The binding energy and variance difference as a function of the bare molecule training iterations is shown in Fig. 1. The obtained variance difference as a function of training iteration is not completely monotonic due to the stochastic optimisation procedure.

FERMIONIC NEURAL NETWORKS

The many-body functions $\psi_i^{k\chi}$, which enter into FermiNet determinants are represented by a deep neural network architecture. Electron-nuclear and electron-electron coordinates are input to the network. The vector separations and their norms are used as inputs: the norm provides the necessary discontinuity to allow the – otherwise smooth – network to satisfy the Kato cusp conditions [2]. We write these inputs as

$$\mathbf{h}_i^{1\chi} = \left(\mathbf{r}_i^\chi - \mathbf{R}_I, \left\|\mathbf{r}_i^\chi - \mathbf{R}_I\right\| \quad \forall \quad I\right) \tag{1}$$

$$\mathbf{h}_{ij}^{1\chi\xi} = \left(\mathbf{r}_i^\chi - \mathbf{r}_j^\xi, \left\|\mathbf{r}_i^\chi - \mathbf{r}_j^\xi\right\|\right), \tag{2}$$

with the notation in brackets indicating the concatenation of the vector separation and its norm for all pairs of indices, $\mathbf{R}_I$ referring to nuclear co-ordinates, χ and ξ labelling fermionic species as in the main text, and $||.||$ the Euclidean norm.

Consecutive transformations update these input features,

$$\mathbf{h}_i^{l+1\chi} = \tanh\left(\underline{\underline{\mathbf{V}}}^l \mathbf{j}_i^{l\chi} + \mathbf{b}^l\right) + \mathbf{h}_i^{l\chi} \tag{3}$$

$$\mathbf{h}_{ij}^{l+1\chi\xi} = \tanh\left(\underline{\underline{\mathbf{W}}}^l \mathbf{h}_{ij}^{l\chi\xi} + \mathbf{c}^l\right) + \mathbf{h}_{ij}^{l\chi\xi}. \tag{4}$$

where

$$\mathbf{j}_i^{\ell\chi} = \text{concatenate}\left(\mathbf{h}_i^{\ell\chi}, \mathbf{H}_i^{\ell\chi}\right) \tag{5}$$

and

$$\mathbf{H}_i^{\ell\chi} = \text{concatenate}_\xi\left(\frac{1}{n^\xi}\sum_{j=1}^{n^\xi}\mathbf{h}_j^{\ell\xi}, \frac{1}{n^\xi}\sum_{j=1}^{n^\xi}\mathbf{h}_{ij}^{\ell\chi\xi}\right). \tag{6}$$

The first transformation (one subscripted index) is referred to as the one-electron stream, and the second (two subscripted indices) is the two-electron stream. These are the 'layers' of the network. The linear dimension of the weight matrices $\underline{\underline{\mathbf{V}}}^l$ and $\underline{\underline{\mathbf{W}}}^l$ determine the 'width' of the layers. These widths, and the total number of layers L, are the hyperparameters which determine the network architecture.

The outputs from the Lth transformation are subject to a final, spin-dependent, linear transformation and multiplied by (in open boundary conditions) an exponentially decaying envelope [3], which enforces the decay of the wave function as $\mathbf{r}_i \to \infty$,

$$\psi_i^{k\chi}(\mathbf{r}_j, \{\mathbf{r}_{/j}\}) = \phi_i^{k\chi}(\mathbf{r}_j, \{\mathbf{r}_{/j}\}) f_i^{k\chi}(\mathbf{r}_j) \tag{7}$$

where

$$\phi_i^{k\chi}(\mathbf{r}_j, \{\mathbf{r}_{/j}\}) = (\mathbf{w}_i^{k\chi} \cdot \mathbf{h}_j^{L\chi} + g_i^{k\chi}) \tag{8}$$

and

$$f_i^{k\chi}(\mathbf{r}_j) = \left[\sum_m \pi_{im}^{k\chi} \exp\left(-\sigma_{im}^{k\chi}|(\mathbf{r}_j^\chi - \mathbf{R}_m)|\right)\right]. \tag{9}$$

The functions $\psi_i^{k\chi}$ are the many-body functions used to populate the FermiNet determinants.

An expansion of FermiNet determinants does not require multiplicative coefficients as they can be trivially absorbed into the determinants [3]. Hutter showed that a single determinant of such many-body functions could represent any antisymmetric

function [4]. However, the proof depends upon the construction of discontinuous functions that cannot be represented in practice by a finite network of a reasonable size.

The set of parameters,

$$\theta = \{\underline{\underline{\mathbf{V}}}^l, \underline{\underline{\mathbf{W}}}^l, \mathbf{w}_i^{k\chi}, \mathbf{b}^l, \mathbf{c}^l, g_i^{k\chi}, \pi_{im}^{k\chi}, \sigma_{im}^{k\chi}\}, \tag{10}$$

are all learnable. The electronic functions, $\chi = (\sigma, -)$, are pretrained to the Hartree-Fock orbitals of the bare molecule, even for positronic calculations, according to the procedure outlined in [5]. For a more extensive description of the FermiNet architecture, see Pfau *et al.* [5].

CALCULATING ANNIHILATION RATES WITH VMC

We follow the procedure for the calculation of positron annihilation rates within the VMC framework described in ref. [6], which we repeat here. The dominant process for positron annihilation in molecular bound states is the 2γ annihilation, which is proportional to the expectation value of the spin-projected contact density between positrons and electrons. The 2γ annihilation rate is thus calculated as,

$$\Gamma = \pi r_0^2 c \sum_{i=1}^{N} \frac{\langle\Psi|\, \hat{O}_i^s \delta(\mathbf{r}_i - \mathbf{r}_+)\, |\Psi\rangle}{\langle\Psi|\Psi\rangle} = 100.9 g(0) \tag{11}$$

where $\mathbf{r}_i$ is the position of the ith of N electrons, $\mathbf{r}_+$ is the position of the positron, $\hat{O}_i^s$ is the spin projection operator to the positron-electron singlet for electron i, r_0 is the classical electron radius, c is the speed of light, and the prefactor has been expressed such that the final expression is given in units of ns^{-1}.

The rotationally and translationally averaged pair correlation function $g(r)$ is calculated as

$$g(r)dr = \frac{1}{2}\mathrm{E}_{\mathbf{r}\sim|\Psi|^2}\left[\frac{\sum_{i=1}^{N} \delta\left(|\mathbf{r}_i - \mathbf{r}_+| - r\right)}{4\pi r^2}\right] \tag{12}$$

where the prefactor of $1/2$ enacts the averaging over spin species resulting from the action of the spin projection operator.

In practice this is accumulated by sampling electron and positron positions from $|\Psi|^2$ via the Metropolis-Hastings algorithm. Electron-positron separations are accumulated into fixed-width bins, which are then normalized to produce a histogram of values of $g(r)$. We fit $\log g(r)$ to a fifth-order polynomial via the Levenberg-Marquadt algorithm as implemented in `SciPy`, excluding bins with zero counts, and extrapolate the value to $r = 0$ to evaluate $g(0)$. The first-order term in the polynomial is fixed to $-x$ to satisfy the Kimball cusp conditions [7]. Extrapolation is necessary due to the vanishing volume of the spherical bins for separations approaching $r = 0$. Errors in the bin counts are estimated as

$$\sigma_r \simeq \sqrt{C_r} \tag{13}$$

where C_r is the number of counts in a bin centered at r. The error in $g(0)$ is obtained from the variance of the 0th order parameter of the polynomial fit.

EXPERIMENTAL SETUP

Four A100 GPUs were used for all calculations presented, except the Benzene molecule, for which sixteen A100 GPUs were used. Single-precision floating point numbers were used in all calculations. We have repeated inference for the positronic ground-state energies of the beryllium oxide (at equilibrium geometry) and benzene molecules in both single and double precision to verify that the final reported energies in the paper are not influenced by the use of single precision floating point numbers. As shown in Table II, any numerical inaccuracy resulting from the use of single precision is not distinguishable within the margin of statistical accuracy we report for any of the values in the main text. This does not discount the possibility that the use of single precision harms the optimization of the wavefunction, but does guarantee that the final results presented in the main text are accurate.

The FermiNet was implemented using the JAX Python library [8], extending a development version of the FermiNet [9]. Optimization used a JAX implementation of the Kronecker-factored approximate curvature (KFAC) gradient descent algorithm [3, 10, 11].

Monte Carlo moves are accepted/rejected on a per-species basis, including spin species. All particles of a given species are moved simultaneously within each Monte Carlo step. Each species has an independent Monte Carlo step proposal size, and this step size is adapted during training to maintain a $\sim 50\%$ acceptance rate.

Kind	Parameter	Value
Network	Number of determinants	32
Network	One-particle stream width	512
Network	Two-particle stream width	32
Optim	Batch size	4096
Optim	Training iterations	3e5
Optim	Pretraining iterations	1e4
Optim	Learning rate	$(1e4+t)^{-1}$
Optim	Local energy clipping	5.0
KFAC	Momentum	0
KFAC	Covariance moving average decay	0.95
KFAC	Norm constraint	1e-3
KFAC	Damping	1e-3
MCMC	Steps between parameter updates	10

TABLE I. FermiNet hyperparameters for all experiments in the paper. For the Benzene calculations, the decay of the learning rate was disabled (i.e. the value was fixed at 1e-4 throughout training)

Molecule	Floating point precision	Ground-state energy [Hartrees]
Benzene	Single	-232.2230(3)
Benzene	Double	-232.2227(3)
BeO	Single	-89.93076(6)
BeO	Double	-89.93081(6)

TABLE II. Comparison of positronic ground-state energies calculated from inference using single and double precision floating point values, demonstrating agreement within statistical error bars.

We found the convergence of the Benzene calculations was accelerated when the decay of the learning rate (see Table I) was disabled. We found that following this aggressive training regime with an additional 2000 training iterations with the appropriately decayed learning rate greatly improved the energies obtained during inference. Understanding the training dynamics of the FermiNet continues to be an open question of great interest.

[1] Flyvbjerg, H. & Petersen, H. G. Error estimates on averages of correlated data. *J. Chem. Phys.* **91**, 7 (1989).

[2] Kato, T. On the eigenfunctions of many-particle systems in quantum mechanics. *Comm. Pure Appl. Math.* **10**, 151–177 (1957). URL `http://doi.wiley.com/10.1002/cpa.3160100201`.

[3] Spencer, J. S., Pfau, D., Botev, A. & Foulkes, W. M. C. Better, Faster Fermionic Neural Networks. *arXiv:2011.07125 [physics]* (2020). 2011.07125.

[4] Hutter, M. On representing (anti) symmetric functions. *arXiv:2007.15298* (2020).

[5] Pfau, D., Spencer, J. S., Matthews, A. G. D. G. & Foulkes, W. M. C. *Ab Initio* solution of the many-electron Schrödinger equation with deep neural networks. *Phys. Rev. Research* **2**, 033429 (2020).

[6] Simula, K. A., Muff, J. E., Makkonen, I. & Drummond, N. D. Quantum Monte Carlo study of positron lifetimes in solids. *Phys. Rev. Lett.* **129**, 166403 (2022). 2202.13204.

[7] Kimball, J. C. Short-range correlations and electron-gas response functions. *Phys. Rev. A* **7**, 1648–1652 (1973).

[8] Bradbury, J. *et al.* JAX: composable transformations of Python+NumPy programs. `http://github.com/google/jax` (2018).

[9] Pfau, D. & Spencer, J. Ferminet JAX implementation. `http://github.com/deepmind/ferminet` (2020).

[10] Martens, J. & Grosse, R. Optimizing Neural Networks with Kronecker-factored Approximate Curvature. *PMLR* **37**, 2408–2417 (2015). URL `https://proceedings.mlr.press/v37/martens15.html`.

[11] Botev, A. KFAC JAX implementation. `https://github.com/deepmind/deepmind-research/tree/master/kfac_ferminet_alpha` (2020).

DATA TABLES

Lithium hydride

	Energy [Hartrees]			
Bondlength [a_0]	Bare	σ_{Bare}	Positronic	$\sigma_{\text{Positronic}}$
2.815	-8.069021	0.000004	-8.100914	0.000004
2.882	-8.069882	0.000005	-8.102727	0.000009
2.948	-8.070345	0.000003	-8.104151	0.000010
3.015	-8.070507	0.000004	-8.105283	0.000004
3.082	-8.070371	0.000007	-8.106226	0.000007
3.148	-8.069958	0.000003	-8.106885	0.000005
3.215	-8.069331	0.000004	-8.107324	0.000003
3.281	-8.068203	0.000278	-8.107583	0.000003
3.348	-8.067207	0.000287	-8.107737	0.000004
3.415	-8.066352	0.000007	-8.107712	0.000011
3.481	-8.065065	0.000005	-8.107542	0.000004
3.548	-8.063663	0.000005	-8.107234	0.000005
3.615	-8.062156	0.000004	-8.106949	0.000006
3.682	-8.060571	0.000004	-8.106551	0.000008
3.749	-8.058894	0.000008	-8.105952	0.000010
3.816	-8.057171	0.000005	-8.105373	0.000132
3.883	-8.055407	0.000007	-8.104972	0.000006
3.950	-8.053575	0.000007	-8.104321	0.000010
4.017	-8.051748	0.000008	-8.103593	0.000004
4.084	-8.049849	0.000008	-8.102936	0.000005
4.151	-8.047965	0.000005	-8.102246	0.000010

Beryllium oxide

	Energy [Hartrees]			
Bondlength [a_0]	Bare	σ_{Bare}	Positronic	$\sigma_{\text{Positronic}}$
		Singlet		
2.115	-89.845004	0.000024	-89.865185	0.000027
2.215	-89.875412	0.000024	-89.896963	0.000019
2.315	-89.893706	0.000014	-89.916586	0.000019
2.415	-89.903115	0.000042	-89.927073	0.000033
2.515	-89.905721	0.000014	-89.930761	0.000057
2.615	-89.903574	0.000075	-89.929260	0.000088
2.715	-89.897711	0.000070	-89.924101	0.000020
2.815	-89.889610	0.000022	-89.915860	0.000043
2.915	-89.879387	0.000073	-89.906657	0.000029
3.015	-89.869008	0.000073	-89.895173	0.000032
3.115	-89.857212	0.000018	-89.884496	0.000039
3.215	-89.846269	0.000021	-89.872785	0.000118
3.315	-89.838491	0.000082	-89.860431	0.000095
3.415	-89.830544	0.000024	-89.845536	0.000027
3.515	-89.820883	0.000027	-89.834588	0.000032
3.615	-89.812532	0.000032	-89.824085	0.000029
3.715	-89.805062	0.000019	-89.812757	0.000077
3.815	-89.798934	0.000097	-89.801516	0.000026
3.915	-89.786483	0.000030	-89.792682	0.000042
4.015	-89.794044	0.000051	-89.794320	0.000043
		Triplet		
3.515	-89.824472	0.000018	-89.822049	0.000031
4.015	-89.784895	0.000023	-89.788294	0.000031
4.515	-89.754943	0.000024	-89.763941	0.000031
5.015	-89.738802	0.000023	-89.745154	0.000032
5.515	-89.732889	0.000022	-89.736450	0.000033
6.015	-89.731046	0.000022	-89.732024	0.000033
6.515	-89.730538	0.000023	-89.730718	0.000034
7.015	-89.730307	0.000022	-89.731238	0.000031
7.515	-89.730150	0.000021	-89.731904	0.000030
8.015	-89.730040	0.000021	-89.731992	0.000036
8.515	-89.730035	0.000025	-89.732091	0.000031
9.015	-89.729969	0.000022	-89.732011	0.000030

Benzene

System	$\langle E \rangle$ [Hartrees]	σ_E^2
Positronic @ 300k iter.	-232.223293	0.000847
Bare @ 134k iter.	-232.216986	0.001202
Bare @ 138k iter.	-232.217466	0.001170
Bare @ 150k iter.	-232.217369	0.001040
Bare @ 176k iter.	-232.219174	0.000871
Bare @ 200k iter.	-232.221368	0.000639
Bare @ 226k iter.	-232.222084	0.000747
Bare @ 240k iter.	-232.222453	0.000568
Bare @ 260k iter.	-232.223825	0.000680
Bare @ 276k iter.	-232.223786	0.000576
Bare @ 300k iter.	-232.225837	0.000668
